# Use of designed sequences in protein structure recognition

**DOI:** 10.1186/s13062-018-0209-6

**Published:** 2018-05-09

**Authors:** Gayatri Kumar, Richa Mudgal, Narayanaswamy Srinivasan, Sankaran Sandhya

**Affiliations:** 1 Lab 103, Molecular Biophysics Unit, Indian Institute of Science, Bangalore, Karnataka 560012 India; 2 Present address: Institute for Research in Biomedicine (IRB), Parc Cientific de Barcelona, C/ Baldiri Reixac 10, 08028 Barcelona, Spain

**Keywords:** Structure recognition, Fold-assignment, Sequence-structure gap, Structural domain assignment, Function annotation, Homology detection

## Abstract

**Background:**

Knowledge of the protein structure is a pre-requisite for improved understanding of molecular function. The gap in the sequence-structure space has increased in the post-genomic era. Grouping related protein sequences into families can aid in narrowing the gap. In the Pfam database, structure description is provided for part or full-length proteins of 7726 families. For the remaining 52% of the families, information on 3-D structure is not yet available. We use the computationally designed sequences that are intermediately related to two protein domain families, which are already known to share the same fold. These strategically designed sequences enable detection of distant relationships and here, we have employed them for the purpose of structure recognition of protein families of yet unknown structure.

**Results:**

We first measured the success rate of our approach using a dataset of protein families of known fold and achieved a success rate of 88%. Next, for 1392 families of yet unknown structure, we made structural assignments for part/full length of the proteins. Fold association for 423 domains of unknown function (DUFs) are provided as a step towards functional annotation.

**Conclusion:**

The results indicate that knowledge-based filling of gaps in protein sequence space is a lucrative approach for structure recognition. Such sequences assist in traversal through protein sequence space and effectively function as ‘linkers’, where natural linkers between distant proteins are unavailable.

**Reviewers:**

This article was reviewed by Oliviero Carugo, Christine Orengo and Srikrishna Subramanian.

**Electronic supplementary material:**

The online version of this article (10.1186/s13062-018-0209-6) contains supplementary material, which is available to authorized users.

## Background

Despite substantial growth in the protein structure database (Protein Data Bank - PDB), contributed by improvements in structural genomics approaches [1] and recently by Cryo-EM techniques [2], we still observe that the number of available structures for proteins is limited. Though it has been over five decades since the advent of X-ray crystallography, unavailability of structures for many protein sequences remains a daunting problem, creating a bottle-neck in function annotation [3, 4]. The minuscule addition of new structural folds in the recent years implies that most protein sequences, of yet unknown structure, have a high probability of adopting one of the many structural folds already known [5]. Added to these constraints, the advent of affordable and high fidelity proteomic sequencing methods has widened the existing gap [6, 7].

Since decades, the sequence-structure-function paradigm has led many to pursue the “holy grail” of structure recognition from sequence [8, 9] with the development of many computational strategies addressing key determinants of folding [10–12]. With advances in computational protein design, sequences have been designed for various applications [13] including probing the sequence space with the end objective of fold recognition [12, 14, 15]. Iterative sequence-profile driven searches [16, 17] or profile- based search routines [18–20] have been shown to be sensitive in homology detection.

Inability to recognize distant evolutionary relationships between proteins may arise from limitations in transitivity of the sequence space, as a consequence of poor sequence dispersion [21]. It has been shown that the sequence space for a naturally occurring domain family can be expanded artificially by computationally generating sequences using the representative PSSM (Position-Specific Scoring Matrix) profiles [21]. Inclusion of such sequences was seen to effectively improve detection of domain family members.

As an improvement over the undirected design approach, sequences were designed between families within a fold, using profile alignments for every possible family pair within a fold [22]. In the absence of such “stepping-stones”, search methods that employ PSSMs [23] and Hidden Markov models [24] are unable to make distant structural associations. There are multiple approaches that identify putative structures for sequences with no detectable homolog, such as threading-based approaches [25–28].

In this study, we use the designed sequences in addition to natural protein sequences to aid in homology detection [29]. The Pfam database [30] clusters sequences into domain families based on conserved functional motifs and sequence similarities, making large-scale functional annotation tractable. The conserved block of residues representing each family was extracted and systematically searched against the designed sequence-enriched space in order to infer structure through ‘homology’. It has been shown earlier that the use of designed sequences in conjunction with four different methods has enabled annotation for 614 DUFs (Domains of Unknown Function) reliably [31]. Through our approach, we were able to provide structural cues for 1392 Protein families (Pfam) of currently unknown structure, of which 423 were DUFs.

Before we applied our approach to recognize folds, we assessed the performance of our method on a dataset of 4058 fold-associated families. For 3993 of these families, a fold was recognized by our approach and fold assignments for 3506 families are found to be correct, yielding a success rate of about 88%. Encouraged by these findings, we extended the approach to the sequence families for which there is no structural information available.

## Methods

### Directed sequence design and search database

Natural linker sequences, which are intermediately related to two distantly related proteins, facilitate homology detection in routinely employed sequence search methods. As described in an earlier publication [22], the paucity of natural linkers in the protein sequence space renders homology detection methods ineffective. To overcome this limitation, an approach to populate gaps in the search space, by purposefully designing protein-like linker sequences between all known families of protein folds provided in the SCOP (Structural Classification of Proteins) database [32] was developed earlier [22]. Briefly, in this approach, each protein domain family, for every known fold in the SCOP database, was represented as a collection of profiles. HMM-HMM alignments were performed between related protein families to generate a combined model that captures the inherent preferences and frequencies of residues between the aligned families. A roulette-wheel based approach was then employed to select for preferred residues at each position in the alignment between every related protein family pair. When repeated along the length of the alignment, the approach generated an ‘artificial linker’ sequence that meaningfully incorporated the observed residue propensities between the aligned families. Using this directed design approach, 3611010 designed sequences were generated between 3901 families for 374 folds in the SCOP database [32]. They are individually available as stand-alone downloadable flat files in the NrichD server [29] for use in tandem with any sequence search procedure.

### Query dataset

The database of sequence families (Pfam 30) [30] are grouped based on sequence similarity into 16306 protein families in the Pfam database corresponding to 1293837 seed sequences. The domains corresponding to the protein families are represented by a multiple sequence alignment, which constitutes the seed sequences. To retain only a representative set, blastclust was applied to the members of each family, at 60% sequence identity and 90% sequence length coverage, decreasing the number of sequences representing all the Pfam families to 234727.

**Fig. 1:**
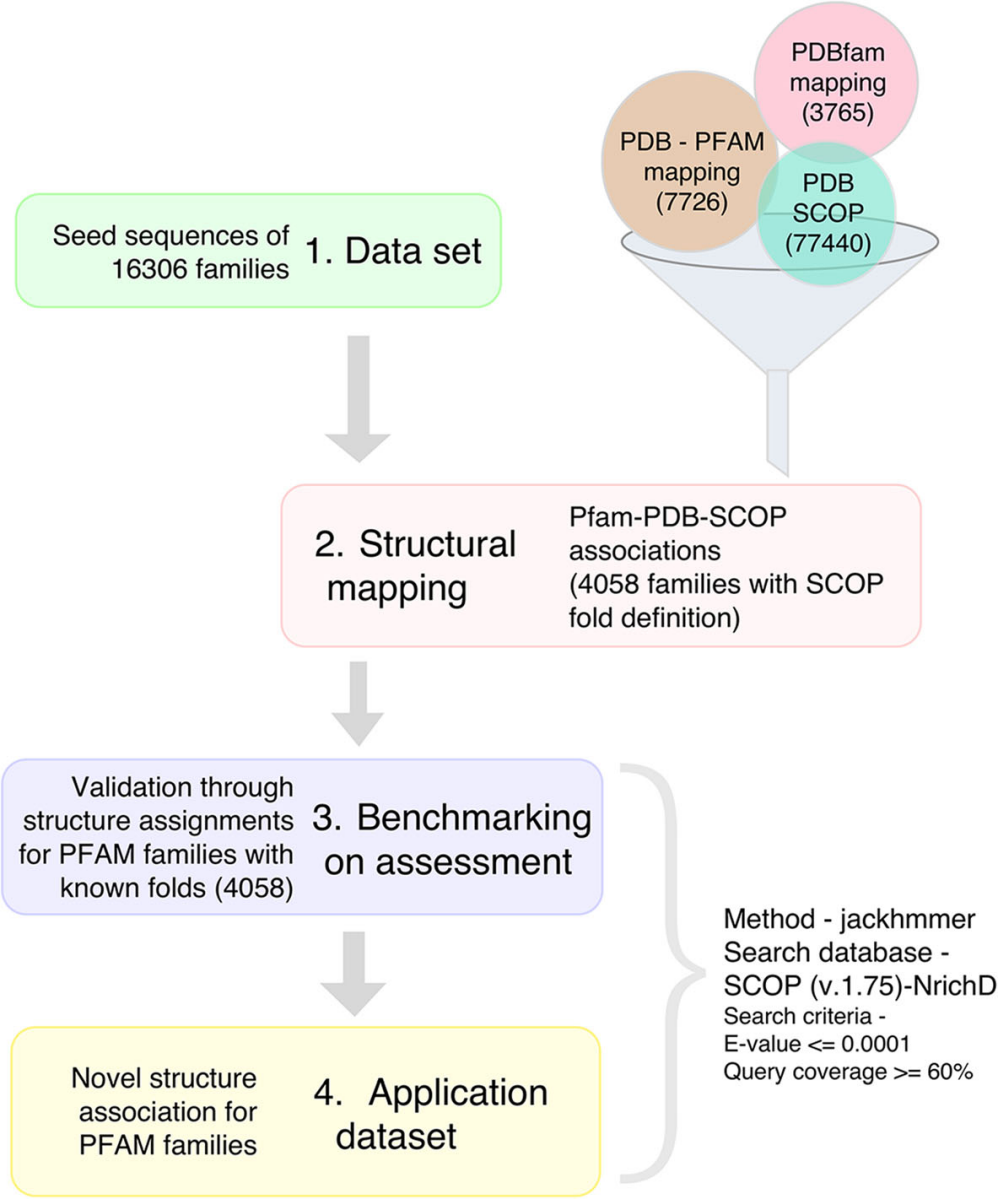
Schematic outline of the workflow: Protocol adopted for structure recognition of families of unknown structure. A consensus was drawn from the structural mapping for the sequence families provided by Xu and Dunbrack [34] and PDB to Pfam mapping available in Pfam [30]

Fold association for PFAM domains is not always direct since multiple SCOP domains may be associated with a single sequence domain and *vice versa*. To identify the SCOP domain associations for various PFAM families, we have pooled together PFAM-SCOP associations by integrating a number of datasets. Firstly, we have used the available SCOP domain definitions for each protein of known structure associated with a PFAM entry based on the PDB id(s), as provided in the SCOPe 2.06 [33] database. Secondly, the RCSB has developed a process, based on the HMMER web service, that takes the PDB-Pfam mappings from SIFTS [34] and adds additional mappings to them [35, 36]. This is provided on the RCSB resource as a downloadable file. Thirdly, academic resources such as PDBfam contain PFAM annotations for ~ 99.4% of chains with more than 50 residues [37]. As shown in Fig. 1, the pooled associations of PFAM-PDB-SCOP from the three resources resulted in 4058 fold associations out of 7726 Pfam sequence families with known structure.

Based on our association of Pfam domain families to the SCOP structural domains, our dataset was divided into two sets: “Assessment” set corresponding to Pfam families for which structural (and fold) association is available and “Application” set corresponding to families for which no structure association is currently available.

#### Assessment set

7726 sequence families were associated with structures and for 4058 families SCOP fold definitions were available for the assigned regions. We considered structural domain associations given in Pfam and PDBfam [34] with an additional condition of better than 60% length coverage of the SCOP domain in order to exclude indiscriminate or false structural associations (Additional file 1: Table S1). These formed the ‘known’ structural associations and were employed to test the strength of our approach. Clans group related protein families together, constituting sequentially divergent families that share common evolutionary ancestry. There are 595 Clans in Pfam 30. The deduction of structure for any one member of the clan translates to the structure and consequently fold association to the other families in the clan [30]. The number of families in each clan ranges from 2 to 254.

#### Application dataset

The remaining 8580 families that had no structure association available were examined for structure recognition at the fold level by extracting the seed sequences from the alignment. We took one representative query sequence per cluster (blastclust) from each family iteratively, until we found hits in our database using jackhmmer [24], at the parameters used for the assessment set.

### Search method: Evaluation and assessment

The workflow has been illustrated schematically in Fig. 1. We employed a sensitive homology detection program, rejuvenating it further by providing a sequence database constituting both natural and designed sequences [29]. This search database, that integrates 3611010 designed sequences with 4694921 natural sequences is available as a resource on the NrichD database as **SCOP(v1.75)-NrichD** with a total of 8305931 sequences. The search algorithm employed, jackhmmer, is a profile-based iterative sequence search method that builds an HMM (Hidden Markov Model) [24] after the first search and uses it as the query in the successive iterations, re-encoding it after every round. We set an E-value filter of 10^−4^ for the reported hits and a maximum of 5 iterations while ensuring the least incidence of profile drifting by making certain that the query protein is present in each iteration. The sequence domain may be associated with single or multiple structural domains corresponding to the same or different structural folds. We minimized cases wherein an equivalent stretch of a sequence domain was associated with different SCOP folds using strict sequence length coverage filters. For assessing the performance of our approach, the families in the “Assessment set” were considered. We quantified the significance of our approach by measuring precision, sensitivity and specificity and identifying criteria to maximize them. These are statistical measures of performance and are represented by the following equations:

$$ \mathrm{Sensitivity}\ \left(\mathrm{Recall}/\mathrm{TPR}\right)=\mathrm{TP}/\mathrm{P}=\frac{TP}{\left( TP+ FN\right)}\times 100 $$

$$ \mathrm{Specificity}\left(\mathrm{TNR}\right)=\mathrm{TN}/\mathrm{N}=\frac{TN}{\left( TN+ FP\right)}\times 100 $$

$$ \mathrm{Precision}\ \left(\mathrm{Positive}\ \mathrm{predictive}\ \mathrm{value}\right)=\frac{TP}{\left( TP+ FP\right)}\times 100 $$

$$ \mathrm{Mathews}\ \mathrm{correlation}\ \mathrm{coefficient}\ \left(\mathrm{MCC}\right)=\frac{TP\times TN- FP\times FN}{\sqrt{\left( TP+ FP\right)\left( TP+ FN\right)\left( TN+ FN\right)\left( TN+ FP\right)}} $$

For a given query Pfam family, the number of correct fold associations that qualify the imposed thresholds are quantified as TP (True positive) while those that fail are designated as FN (False negative). Similarly, for a given query Pfam family, the number of incorrect fold associations that qualify the imposed thresholds are designated as FP (False positive) while those which are not hits from folds other than the correct fold are considered as TN (True negative).

For each Pfam family, based on the folds of the hits obtained through jackhmmer searches, a SCOP fold is associated with the query sequence. To parse the results obtained for sequence families with no previously known structure, the criteria as determined from the assessment were query length coverage at better than 60% and E-value better than 10^−4^. In addition, further constraints were added to exclude false positives. For the Assessment dataset, we observed that the correct fold was associated with the highest normalized frequency for a given query.

Normalized fold frequency is given by $\frac{fold(i)}{N},i\in \left[1,n\right].$

where *n* is the total number of folds associated with a query sequence and fold(*i*) represents the number of homologues identified from that fold in the profile search. *N* is the total number of associations across folds for the query.

Based on the above observation, using normalized fold frequency, we could further rank the associations in our Application dataset as –

Confident* - If the fold with the highest frequency also had an association at greater than or equal to 95% query coverage.

**Confident** – If the fold with the highest frequency provides the best coverage between 60 and 95%.

**Conflict** – When the highest fold frequency did not give the best query coverage.

**No ambiguity** - If there is only a single structural fold associated with a query, we consider the association made at best query coverage.

## Results and discussion

### Assessment: Revisiting families of known structure

Structural fold associations are available for 4058 families *a priori*. A sequence representative of each family was queried against the database. Of the 4058 fold-associated families, we were able to assign folds for 3993. We observed that out of the 3993 families, for 3506 associations were made correctly, satisfying stringency filters specified by us (see Methods section). For 487 families, incorrect associations were made at 60% and better query coverage. As discussed in the Methods section, we analyzed these false positives and identified additional criteria to minimize the occurrence of false positives. We have used the revised criteria while recognizing folds for Pfam families of unknown structure. Of the 3993 families for which we made structural associations, 2603 corresponded to 467 Clans and by extension the structural domain can be associated with other Clan members, corresponding to ‘inferred’ structural association.

**Fig. 2:**
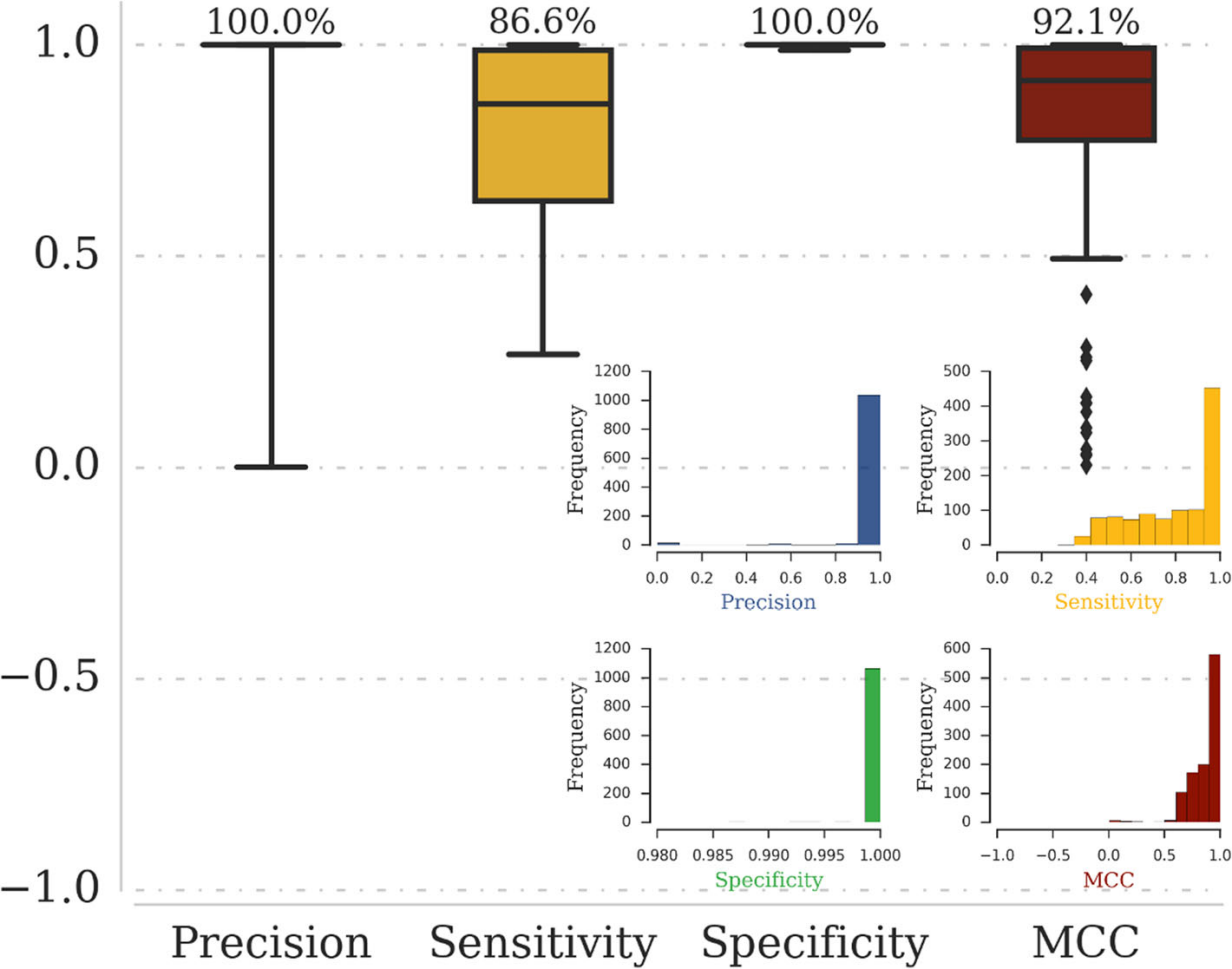
Evaluation of the approach: The precision, sensitivity and specificity of results from the assessment dataset are represented and the median value for the respective distributions is indicated above each boxplot, in percentage. The inset figure shows the histogram representing the frequency of each interval for the assessment dataset. Query coverage cutoffs of greater than 60% and E-value thresholds of better than 10^−4^ were used

We evaluated the performance metrics by determining the precision, sensitivity and specificity measures. In Fig. 2, the boxplots show the precision, sensitivity, specificity and MCC distribution. Our approach performs well at query coverage better than 60% and E-value better than 10^−4^, as indicated by the median values of 100% for precision and specificity and 86.6%% for the sensitivity. MCC values for the families in our dataset (range lies between 0 and 1) show that our search protocol performs reasonably well for most of the queries evaluated here. The histogram for the performance metrics is shown as individual distributions in the inset of Fig. 2. The frequency distribution of incorrect vs. correct association as a function of query coverage highlights the importance of excluding associations at lower coverage at the expense of a few false negatives (Additional file 2: Figure S1)

We have also evaluated the performance of our method as a function of query length, secondary structure classes and repeat-containing folds (Additional file 3: Figure S2). We find that sensitivity is the only parameter which shows a crude relationship to the query length, while the performance of the method is independent of all the other parameters assessed here. Also, we observe that sensitivity of the search protocol is, in general, comparable for the protein queries from various classes except the α/β class. For peculiarities owing to amino acid compositions, we have identified protein folds that are known to contain structural repeats in our assessment dataset and examined the performance of our fold assignments for such cases with respect to other protein folds. We find that the values of assessment parameters are quite similar between folds with repeats and other folds.

**Fig. 3:**
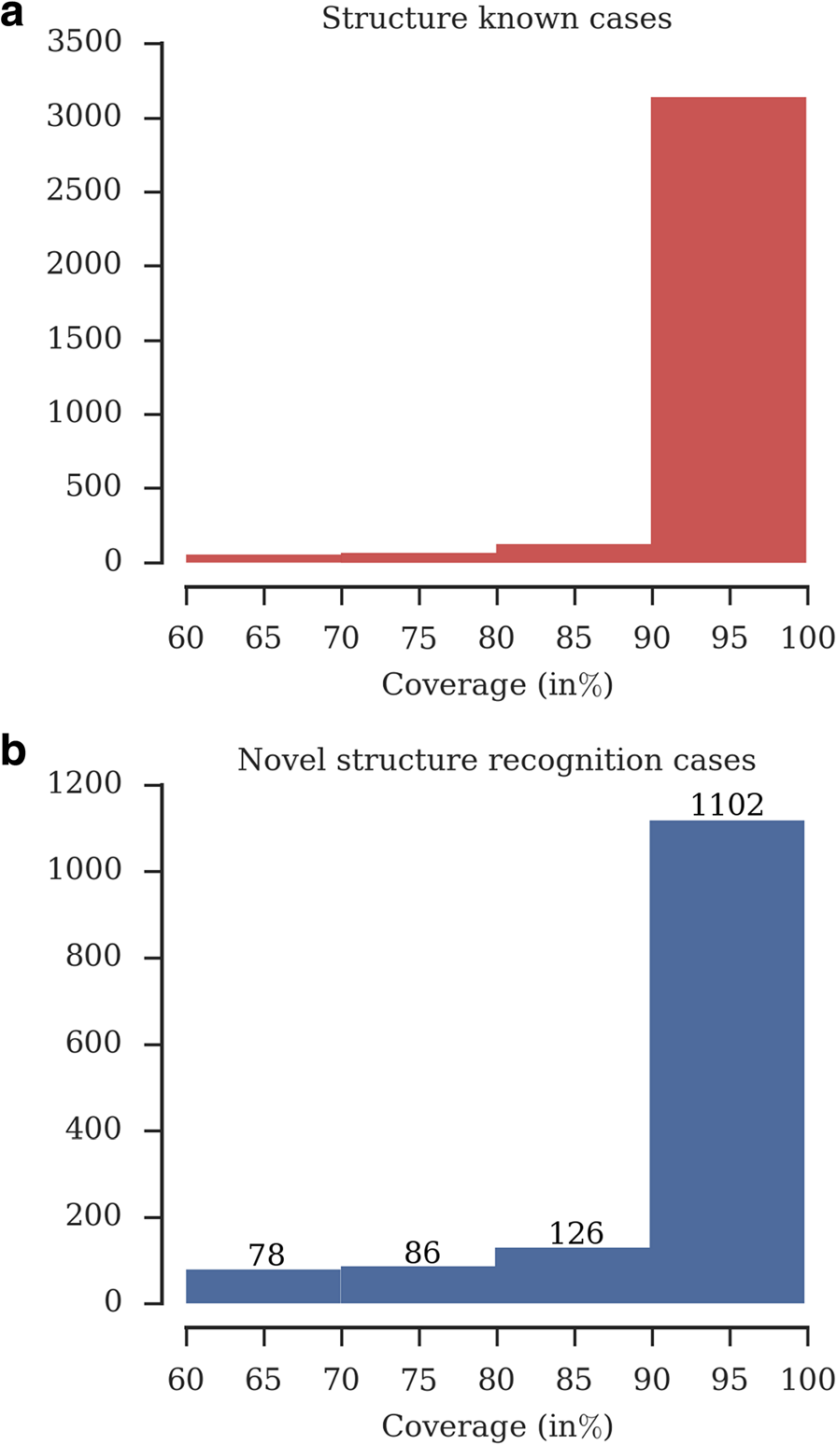
The frequency of structural fold associations for sequence families as a function of coverage made in the searches: **a**) families with structure and fold information available. **b**) families with no prior structure information associated with a structural fold by our approach

The tabulated details on individual families in the assessment of our approach are provided, indicating the sequence details in addition to the strength of the assignment (E-value and coverage in Additional file 4: Table S2). For 3257 families, we made the correct fold associations at greater than 90% query coverage (Fig. 3a). We note that the search is biased towards identifying single domain associations at the given restraints. Undoubtedly, relaxing the strict search criteria would lead to identifying additional structural domains but at the added expense of including false positives.

#### Revising metrics: Minimizing occurrence of false positives

We extended the criteria of better than 60% query coverage and an E-value better than 10^−4^ for the cases of unknown structure, as these conditions were derived from the benchmark analysis of the assessment dataset. Associations to more than one fold for the cases of known structure led us to identify a diagnostic metric, which could help identify the correct structural fold if there are multiple folds reported in the hits. Since homologous proteins adopt the same fold even at low sequence identity, the likelihood of ultimately reporting the correct fold association is enhanced as most of the query sequences in a family are likely to identify hits corresponding to the same fold. We observe that a greater number of positive hits from the correctly related fold are more likely to direct the association to the correct fold (Additional file 5: Figure S3) since they are more likely to direct the search to the correct fold when considered in iterative profile/model-based search procedures. Our analysis of hits, in the jackhmmer searches performed here, shows consistently that the fold with the highest normalized fold frequency most often corresponds to the correct fold association (Additional file 5: Figure S3).

We also examined the cases wherein the query sequence reported a correct and incorrect association and compared the best query and target coverage for the correct and incorrect associations (Additional file 6: Figure S4). We observed a significant difference using chi-squared test for the query coverage as well as target coverage (*p*-value < 10^−5^) for the correct vs. incorrect association. We also observed that the approach performs better as a function of the query and target alignment length (Additional file 6: Figure S4).

### Fold recognition for Pfam families of unknown structure

We were able to make structural assignments for 1392 families for which there are no structures available. Our results are listed in Additional file 7: Table S3. For 1095 families, fold associations have been made at better than 90% query coverage (Fig. 3b). For this set of sequence families with no structural information we increased the stringency filters to ensure the maximum likelihood of identifying the correct fold. We observed from our assessment dataset that the fold associations for 3506 families reported at the highest frequency (normalized fold frequency ≥ 0.8) correspond to the correct fold and deployed this observation (Additional file 5: Figure S3) in the “application” dataset. We also had 88 cases marked as “conflict”, as the fold with the highest frequency did not give the highest coverage in comparison to the other folds identified. Careful analysis of these results showed that these associations were not necessarily incorrect but had lower confidence. For 348 cases, we observed that fold agreement was seen among the clan members, adding credibility to our findings. However, for 158 families the structural fold associated did not concur with other members of the clan, which could imply additional structural domains associated with the clan members. Of the 1392 families, fold associations were made for 423 DUFs of which, in an earlier analysis from this group [31], the same folds were reported for 109 DUFs. For the remaining 314 cases, we report evolutionary relationships for the first time. There were 377 cases wherein we made “Confident*” associations with greater than 95% query coverage. We annotated 181 families as “Confident”, because the fold with highest incidence was concurrently associated with the highest query coverage. 902 cases were marked as “No ambiguity” because all the associations were made with the same fold and the one with the best coverage (greater than 60%) was reported. We also made fold assignments for the families which were associated with PDB entries that lacked SCOP fold annotation. We identified “Confident*” cases and have compared the template identified by us with the structure associated in the Pfam repository using TM-Align [38] and have listed cases with TM-score > 0.5 (Additional file 8: Table S4). The objective of our work was to make the structure assignments easily scalable by using a sequence-driven approach. Structural associations were made for 1392 families, by considering 36908 representative seed sequences. If one were to consider the sequences representing the 90% non-redundant set of Pfam families [39], the number of sequences for which we are able to extend structure assignments escalates to 725696, providing further resolve to our approach.

#### Evaluation of families in Pfam 31 with structure and fold annotation available

When our work was completed a new Pfam version (Pfam 31) was released. Pfam 31 has fold associated for part or full-length of 20 families included in our application set. To verify if the fold annotation that is presently available for 20 families agreed with our results, we compared the associated folds with those reported by our approach. For 18 families a consensus was observed, while for two cases, our assignments differed. We examined the results and found that the correct folds were reported as hits for the two families in the jackhmmer searches as well. However, they were not ultimately reported in our study, as they do not correspond to the highest normalized fold frequency. The details of the associations are provided in Additional file 9: Table S5.

## Conclusions

The gold standards for structural annotation and biochemical characterization are protein crystallography, NMR, Cryo-EM and laboratory biochemical studies, however, they are time consuming [9]. Consequently, a significant number of structure un-annotated sequences are available in sequence databases. Though high sequence identity between sequences is a strong correlate for homology implying similar structure and function, members of a structural fold can often share very low sequence similarity [40–42]. Hence, one can expect that a sequence may adopt the same structural fold as a distant structural neighbor. As structure drives the function [43], the ability to assign a tentative structural fold to a protein family as a structural approximate makes functional annotation a realistic goal for the families of yet unknown function.

In this work, we report folds for 1392 protein families of unknown structure. In our approach we have combined the strength of an iterative HMM-based search method [24] and a sequence database enriched with computationally designed ‘linker’ sequences. Such linker sequences are poised to function as connectors between protein sequences in families that have undergone large sequence divergence. Especially, they are observed to link and detect remote protein relationships when natural linkers between such proteins are unavailable. In recent years, several refined and rigorous search methods have been made available to recognize such relationships and indeed they are becoming increasingly powerful. However, they all rely on the ability to detect natural linkers from which they derive the first principles and fingerprints to power their search towards the correct and appropriate structure/function association for a query protein. When there is a paucity of such natural linkers, these search methods face severe limitations. Our approach to design protein sequences fills this gap by using the sequence properties of more than one protein family in a fold to design artificial linkers. Such artificial linkers when plugged into commonly employed search databases reduce the gaps between distant proteins. Indeed, our attempts to associate protein folds to protein families of yet unknown structure is a step in this direction. Comparisons with available search methods such as SUPERFAMILY were able to associate the same fold, as in our assignments for only 292 protein queries. No assignments could be made by SUPERFAMILY for the remaining queries.

The assignment of a fold to a protein query sequence is only a start point for its function annotation since it is well known that there are promiscuous folds, such as the TIM fold that are associated with several functions, and likewise there are functions such as DNA-binding that are associated with more than one fold. Fold assignment, however, enables us to evaluate this in the light of function of known members of the fold. It must be noted that if a novel function, hitherto unreported for the fold, is performed by the protein query/ family in the study, it may not be discovered through an assessment such as ours. We believe that the families for which we assign structures may function as a nodal point in guiding focused experimental efforts. The driving force of our study was to elucidate an entirely sequence-dependent approach for structural recognition by filling the gaps in sequence space. While this has been possible for a large number of families in our dataset, confident associations could not be made for 284 families which were associated with multiple structural domains. Specifically, for Pfam families associated with multiple structural domains, a target coverage greater than 90% could be given precedence over the 60% query length coverage criteria to overcome this limitation. Also, an enhanced search space, in conjunction with other methods can improve the confidence of such assignments leading to breakthroughs in computational structural genomics.

## Reviewers’ comments

### Reviewer’s report 1

Oliviero Carugo, University of Vienna

## Reviewer comments

The manuscript submitted by Srinivasan et al. describes an empirical computational procedure to recognize the fold of a protein based on (i) its sequence, (ii) the sequences of some homologous proteins, and (iii) simulated sequence information. A conservative use of jackhmmer is done and several heuristic filters based on coverage and similarity are introduced to limit the emergence of false positives. It is also interesting the careful use of the Clans. The manuscript is certainly interesting and deserves publication though some modifications seem to be needed.

I have two main questions.

(1a) On the one hand, no comparison is made with other techniques, while this would enrich considerably the manuscript.

Author’s response: *We thank the reviewer for his positive comments and support for the manuscript. Reviewer 2 and 3 have also suggested that we compare the results we obtained with the results of another search method. In response, we have queried sequences from 1392 PFAM families, which are listed in Additional file* *7**: Table S3, against the SUPERFAMILY database. Interestingly, we obtain associations using SUPERFAMILY only for 292 queries. For the remaining 1100 queries we did not get any hits in searches in the SUPERFAMILY. Among the 292 hits, we have agreement in the fold assignment for 265 queries. For 27 cases where there is a disagreement, to resolve these conflicting cases requires more in-depth analysis which we intend to pursue later since it is beyond the scope of the current study. Details of these results are included in the Additional file* *7**: Table S3 as a separate column wherein agreement is marked as “fold match”.*

*However, it must be noted that the main purpose of using the designed sequences is to empower search algorithms in a method-independent manner. The improved sensitivity that we observe for the 1392 queries in our database that includes designed sequences when compared with SUPERFAMILY, as shown in this analysis, reaffirms this. In this project we chose to include the designed sequences in a database of SCOP sequences and their closely-related homologues. This database was searched using jackhmmer. In principle, the designed sequences may be included in any database of natural protein sequences and any homologue search method could be employed. In that sense, our protocol is method- independent.*

(1b) Another interesting question: is the performance of the computational procedure described in this manuscript different on various types of folds (different number of amino acids, different amino acid composition, different secondary structure based class, etc.)? Some additional modifications seem to be necessary to improve the quality of the manuscript.

Author’s response: *Revised manuscript includes a new file (Additional file* *3**: Figure S2), which shows the performance of the search method on various types of parameters suggested by the reviewer (different number of amino acids, different amino acid composition, different secondary structure based class, etc.). Almost all the protein families in our dataset belong to globular folds and, therefore, LCR (Low complexity regions) containing sequences are excluded. However, we assessed our parameters on structure repeats, which might have some peculiarities in amino acid composition which we have included in the supplementary information.*

*In summary, we find that sensitivity is the only parameter with dependence on the query length, while the performance of the method is independent with respect to all the other parameters assessed here. Also, we observe that sensitivity of the search protocol is in general comparable for the protein queries from various classes except the α/β class to an extent. For peculiarities owing to amino acid compositions, we have identified protein folds that are known to contain structural repeats in our assessment dataset and examined the performance of our fold assignments for such cases with respect to other protein folds. We find that the values of assessment parameters are quite similar between folds with repeats and other folds. We have provided this information in the supplementary section in Additional file* *3**: Figure S2 and in the main manuscript on Page number 12.*

(2a) Despite the presence of a list of abbreviations, the manuscript becomes much more readable if the abbreviation is described where it is encountered for the first time. For example, in line 77, “PSSM” should become “PSSM (Position Specific Scoring Metrix)” and “(Position Specific Scoring Matrix” should be removed in line 83. Also, in line 95, “DUF” should become “DUF (Domain of Unknown Function)”, etc.

Author’s response: *We thank the reviewer for drawing our attention to this point and have now corrected it by expanding the first instance of the abbreviations in the manuscript.*

(2b) I think that I understood what is written from line 121 to line 130. However, I am not sure and I needed some imagination to guess the meaning of this paragraph. I strongly encourage the Authors to rewrite is entirely to make it really understandable. I also note that Fig. 1 does not help much.

Author’s response: *We thank the reviewer for his suggestion to revise this section and have now rewritten these lines to improve its readability by including a more detailed description of how folds have been associated with PFAM families in our assessment dataset. We have also modified Fig.* *1* *accordingly to explain the workflow better.*

(2c) I agree with the definitions of sensitivity, specificity and precision, and correctly the Authors define them explicitly, since there is some nomenclature confusion about them. Note, for example, that the specificity is sometime defined, alternatively, as TP/(TP + FP) (Eidhammer, Jonassen, Taylor, Protein Biochemistry, Wiley, 2004). I would suggest the Authors to use also the Matthews correlation coefficient (Mcc), which is probably the most robust figure of merit in this family of figures based on the confusion matrix.

Author’s response: *We have now extended the assessments to include the MCC metric to assess the performance of the designed sequences in detecting distant relationships, in addition to sensitivity, specificity and precision that we had earlier provided. Here, we would like to draw the attention of the reviewer and the readers that the MCC value is predominantly high (median ~ 0.92) for the queries analyzed, suggesting a uniformly high accuracy in distinguishing the true positives from the false positives at the search criteria employed. As suggested by the reviewer in the subsequent point, we have now included the figure pertaining to this in the main manuscript (Fig.* *2**).*

(2d) I also suggest to include most of the supplementary material in the main text, especially tables of sensitivity, specificity and precision etc.

Author’s response: *We thank the reviewer for suggesting that we present the results of our assessment in the main manuscript. We have included a figure (Fig.* *2**) in the main manuscript which shows box plots and histograms for assessment parameters.*

### Reviewer’s report 2

Christine Orengo, University College London

## Reviewer comments

The authors describe a method for assigning protein structures to structurally uncharacterized families in the Pfam database. This exploits HMM based strategies for scanning a dataset of sequences, comprising some designed sequences to increase the ability to explore more of sequence space. They report a compelling level of success using a benchmark of structurally characterized Pfam familes. They also provide predicted structural annotations for 1392 Pfam families of unknown structure. This is an interesting idea and the results are impressive. Sensible strategies have been employed in the protocol developed by the authors eg selecting the fold predicted with highest normalized frequency. I think the article would benefit from some more clarity in places. For example, the methods could be more clearly described in places. It would help to have a summary of how the designed sequences are generated ie it shouldn’t be necessary to read another paper to understand this or the composition of the search database.

Author’s response: *We thank the reviewer for her positive comments and observations on the manuscript. As suggested by the reviewer, in order to improve the clarity of the manuscript in the sections pertaining to the methods employed to design sequences (Mudgal et al., J Mol Biol. 2014 Feb 20;426(4):962–79), we have now included details of the sequence design procedure in the* *Methods* *section in the sub-section titled “****Directed sequence design and search database”****.*

1. It would be helpful to know what proportion of sequences in the search database are designed sequences.

Author’s response: *Of the total 8305931 sequences in the search database* ***SCOP(v1.75)-NrichD,*** *3611010(~ 44%) are sequences that were designed between 374 SCOP folds. We have now also included this detail in the manuscript in a sub-section in the* *Methods* *section titled “****Search method: evaluation and assessment”****.*

2. It would be good to see some results on how well the protocol works without designed sequences in the search database. In their previous work the authors link superfamilies within fold groups and show the value in using designed sequences to increase detection of very remote homologues. However, in this current work they are trying to do something less challenging since Pfam does not aim to recognize very remote homologues, as in SCOP, so some assignments are possibly not very remote homologues and could possibly be obtained using HMM-HMM strategies ie as in HHsearch without designed sequences.

Author’s response: *As rightly pointed by the reviewer, Pfam does not aim to recognize very remote homologues, as in the SCOP database. Therefore, for many proteins that undergo extensive sequence divergence, their inability to find relationships with proteins of known function remains a limiting step in their effective annotation. For all the families for which we propose a new evolutionary relationship in this work, through the searches in a database augmented with designed sequences* ***SCOP(v1.75)-NrichD****, we performed searches in the* ***SCOP(v1.75)-DB*** *that contains 4694921 sequences of sequences of known structures from SCOP and their sequence homologues from UniProt. This database is also available in the NrichD database resource. For 70 queries, we were unable to make any connections when we searched in the database of natural sequences that was devoid of designed sequences. For such queries, searches in the augmented database, which includes artificial sequences, were more rewarding, offering clues to their potential fold, through the use of designed intermediates.*

*As mentioned in our earlier publication (JMB, NAR), the sensitivity for searches made in the SCOP-NrichD database is significantly more than searches made in the SCOP-DB. However, sometimes due to profile drifting, few important hits can be missed out. Therefore, as suggested in our prior publication, both the databases may be queried to provide the results as a union of both searches.*

*The main point of our present work is to propose evolutionary relationships for 1392 families of unknown structure for the first time.*

3. Therefore, it would also be good to see some benchmarking against other approaches assigning structural annotations to Pfam sequences – For example if the authors simply search InterPro or SUPERFAMILY using the representative Pfam sequences how often would they obtain hits providing structural annotations for the families ie using HMM based scans in InterPro.

Author’s response: *We thank the reviewer for raising this point. Reviewer 1 and 3 have also suggested that we compare our results with the results of other search methods. In response, we have queried for sequences from 1392 PFAM families, which are listed in Additional file* *7**: Table S3, against the SUPERFAMILY. Interestingly, we obtain associations only for 292 queries. For the remaining 1100 queries we did not get any hits in the SUPERFAMILY searches. Among the 292 hits, we have agreement in the fold assignment for 265 queries. For 27 cases where there is a disagreement, to resolve these conflicting cases requires more in-depth analysis which we intend to pursue later since it is beyond the scope of the current study. Details of these results are included in the Additional file* *7**: Table S3. Hits obtained in Superfamily searches are indicated as “fold match” where they agree with the associations also made in our searches. We have also mentioned this in the* *Conclusion* *section.*

4. For the 423 DUFs for which they assign putative folds – were any of these DUFs allocated folds in the work of Mudgal et al. and if so do the fold assignments agree with those author’s predictions?

Author’s response: *Of the 423 DUFs with fold association made in the current work, 109 associations overlap with the folds allocated in the work of Mudgal et al. The fold assignment for these 109 cases are identical between the current work and the Mudgal et al. publication. For the remaining 314 cases, we are reporting evolutionary relationships for the first time in our present study. This has been mentioned in the manuscript in the sub-section “****Fold recognition for Pfam families of unknown structure”****.*

5. How many different folds are covered by their structure predictions for Pfam families? ie it would be good to show the distribution of assignments to the different folds predicted. In the past you could do better than random in predicting folds by opting for TIM barrel or Rossmann fold! So it would be very interesting to know if there is still a high probability that uncharacterized sequence families adopt one of these two folds since they do cover a large proportion of sequence space.

Author’s response: *We observe the high frequency folds in our associations are largely the repeat containing folds. In response to Reviewer 1 we carried out a study to assess if the sensitivity and specificity measures for folds containing structural repeats and we do not observe a significant difference with other folds. Among the other high frequency folds, we also see TIM barrel, P-loop containing nucleoside triphosphate hydrolases and ferredoxin-like to name a few.*

6. On that note, how representative of structure space are the folds in their search database? i.e. what proportion of SCOP families and SCOP structures map to these folds? 7. In the abstract, the authors mention that fold assignments for some of the DUFs is a step towards functional annotation. Perhaps this should be expanded as some caution is needed. There is no clear correlation between fold and function. However, knowing the fold could provide some clues on the location of the active site in enzymes permitting mutagenesis studies to elucidate functional residues.

Author’s response: *Our search database is the* ***SCOP(v1.75)-NrichD*** *database which is, in principle, an extension of the SCOP database with sequence homologues from the UniProt database for proteins of known structures for all the folds in the SCOP database. In addition to this spread of sequences, we have augmented the database with designed sequences for as many as 374 folds. Therefore, our database is an extension of SCOP and all the families and folds represented in SCOP are represented in their entirety in our search database as well.*

*Secondly, we completely agree with the assertion of the reviewer that gleaning function from fold is non-trivial. In order to avoid the expectation of function annotation for the 1392 PFAM families in our study, we have removed mention of it from the Abstract and included points pertaining to the underlying difficulties in such an interpretation to the* *Conclusion* *section.*

### Reviewer’s report 3

Srikrishna Subramanian, Institute of Microbial Technology

## Reviewer comments

In their manuscript “Use of designed sequences in protein structure recognition,” Gayatri Kumar et al., describe a workflow wherein they computationally generate novel protein sequences for Pfam families that are known to share the same fold thereby allowing to bridge the sequence space between these families transitively. The method to generate these sequences has already been published earlier by this group, and in this manuscript, the authors use the extended sequence space to assign folds with varying degrees of confidence to Pfam families that currently have no structural representatives. Overall, the project is well executed, and detailed results are provided in five supplementary tables. I don’t have any major comments.

Author’s response: *We would like to thank the reviewer for his positive feedback on our manuscript.*

However, given that this is an exercise in remote homology prediction, I would have liked to see one-on-one comparisons to other well-established tools such as those used routinely in CASP.

Author’s response: *We thank the reviewer for raising this point. Similar question was also raised by Reviewers 1 and 2. In response, we have queried for sequences from 1392 PFAM families, which are listed in Additional file* *7**: Table S3, against the SUPERFAMILY. Interestingly, we obtain associations only for 292 queries. For the remaining 1100 queries we did not get any hits in the SUPERFAMILY searches. Among the 292 hits, we have agreement in the fold assignment for 265 queries. For 27 cases where there is a disagreement, to resolve these conflicting cases requires more in-depth analysis which we intend to pursue later since it is beyond the scope of the current study. Details of these results are included in the Additional file* *7**: Table S3. Hits obtained in SUPERFAMILY searches are indicated as “fold match” where they agree with the associations also made in our searches. We have also mentioned this in the* *Conclusion* *section.*

Also, it would be good to see how the current approach of sequence fill-in between families of the same fold compares to the previously described undirected design approach.

Author’s response: *We have analyzed in depth ~ 110 queries from 11 folds for their ability to pick up homologues through the use of undirected designed sequences that were described in an earlier publication from us (Sandhya et al., Mol.Biosystems, 2012). For this, sequences were designed for each family in the following folds- a.118, c.69, c.66, f.38, c.10, b.82, b.69, b.82, b.3, a.25, d.144, through the undirected sequence design procedure. Select queries from PFAM families that had made associations with a SCOP fold from our application dataset (reported in Additional file* *7**: Table S3) were queried in the* ***SCOP(v1.75)-DB with*** *4694921 sequences that was augmented each time with the undirected designed sequences for that fold. Of the 110 queries, we observed that there are 70 queries that uniquely associated with the SCOP fold only when searched in the* ***SCOP(v1.75)-NrichD*** *database. 28 queries could make associations when also queried in the* ***SCOP(v1.75)-DB*** *that was augmented with the corresponding designed sequences using the undirected approach. This demonstrates that the sensitivity of the search improves nearly three fold on the inclusion of the directed designed sequences as compared to the undirected designed sequences. The design of sequences, purposefully, between all families within a fold (Mudgal et al., JMB, 2014) was shown to be an advancement in our attempts to employ designed sequences as artificial linkers that could facilitate homology detection. Therefore, we have performed the comparative searches using the undirected designed sequences based on the reviewer’s suggestions and report our observations here, but have chosen not to include it in the current manuscript.*

A bit more discussion as to why this method works so well, it potential caveats, etc. would be desirable.

Author’s response: *We thank the reviewer for suggesting changes that would improve appreciation of our approach and have now included these details in the* *Conclusion* *section of the manuscript.*

Also, some insight into why the correct fold assignment is correlated to the highest normalized frequency would be of interest to the readers.

Author’s response: *It is well appreciated that homologous proteins adopt the same fold even at low sequence identity. However, sequence dispersion can be high and limit the ability of sequence search methods to capture such relationships. We believe that when many queries are employed to perform a search or when many hits from the same fold are reported in the search results, the likelihood of ultimately reporting the correct fold association is enhanced. However, due to the inherent sequence dispersion in protein folds, searches need not be unidirectional and a number of false positives are possible. Therefore, we expect that a greater number of positive hits from the correctly related fold are more likely to direct the association to the correct fold and therefore compute the normalized fold frequency to guide our associations. We have included these points on the normalized fold frequency also in the manuscript in the subsection titled “****Revising metrics: Minimizing occurrence of false positives”****.*

## Additional files

Additional file 1: **Table S1.** List of Pfam families and the associated SCOP fold annotations obtained through mapping onto PDB entries. (XLSX 125 kb)

Additional file 2: **Figure S1.** The frequency distribution of sequence query coverage for correct and incorrect fold associations: “Blue” represents the incorrect and “red” the correct associations respectively. The median for the distribution of “incorrect” associations corresponds to 30.13% query coverage, represented by the dotted line. 3.18% of the correct fold associations are to the left of this median value. (PNG 72 kb)

Additional file 3: **Figure S2.** Performance of our approach as a function of different parameters: **a)** Query length – Performance as a function of the number of amino acids, annotated are the points above 0.8 for: Sensitivity (621*), Specificity (1110*), Precision (1077*) and MCC (782*). **b)** Repeat containing folds – Comparative performance of folds in our assessment dataset containing structural repeats with other folds. **c)** Secondary structure based SCOP classes – Performance metrics evaluated across different secondary structure based SCOP classes “a” through “g”, which are as follows: a (All-α), b (All-β), c (α/β), d (α+β), e (Multi-domain proteins), f (Membrane and cell surface proteins and peptides) and g (Small proteins). (PNG 395 kb)

Additional file 4: **Table S2.** List of Pfam family queries with structural fold annotation available and validated in the assessment of our approach. (XLSX 355 kb)

Additional file 5: **Figure S3.** The normalized fold frequency of correct vs. incorrect associations for the assessment dataset: The preponderance of ‘correct’ associated folds (in green) is observed at a higher normalized fold frequency than other ‘incorrect’ fold associations (in red). (PNG 92 kb)

Additional file 6: **Figure S4.** False vs. True positives for queries in the assessment dataset: The distribution of true positives vs. false positives as a function of **a)** Query and target coverage. **b)** Query and target alignment length (number of residues in the alignment). (PNG 363 kb)

Additional file 7: **Table S3.** List of structural associations by our approach: Details of the structural fold associations and the confidence of the assignments for queries from 1372 families is given. (20 families for which structures are available in Pfam 31 have been moved to Table S5). (XLSX 234 kb)

Additional file 8: **Table S4.** TM-Align scores for Pfam families with known structure information but no fold association available in SCOP. (XLSX 14 kb)

Additional file 9: **Table S5.** Pfam 31 families now associated with structural folds and consensus with our application dataset. (XLSX 21 kb)

## Abbreviations

DUF: Domains of Unknown function
HMM: Hidden Markov Model
PDB: Protein data bank
Pfam: Protein family
PSSM: Position-specific scoring matrix
SCOP: Structural Classification of proteins
