# Supplementary figures and images for: Use of designed sequences in protein structure recognition
Source: Biol Direct. 2018 May 9;13:8. doi: 10.1186/s13062-018-0209-6 (PMC5960202; doi:10.1186/s13062-018-0209-6)

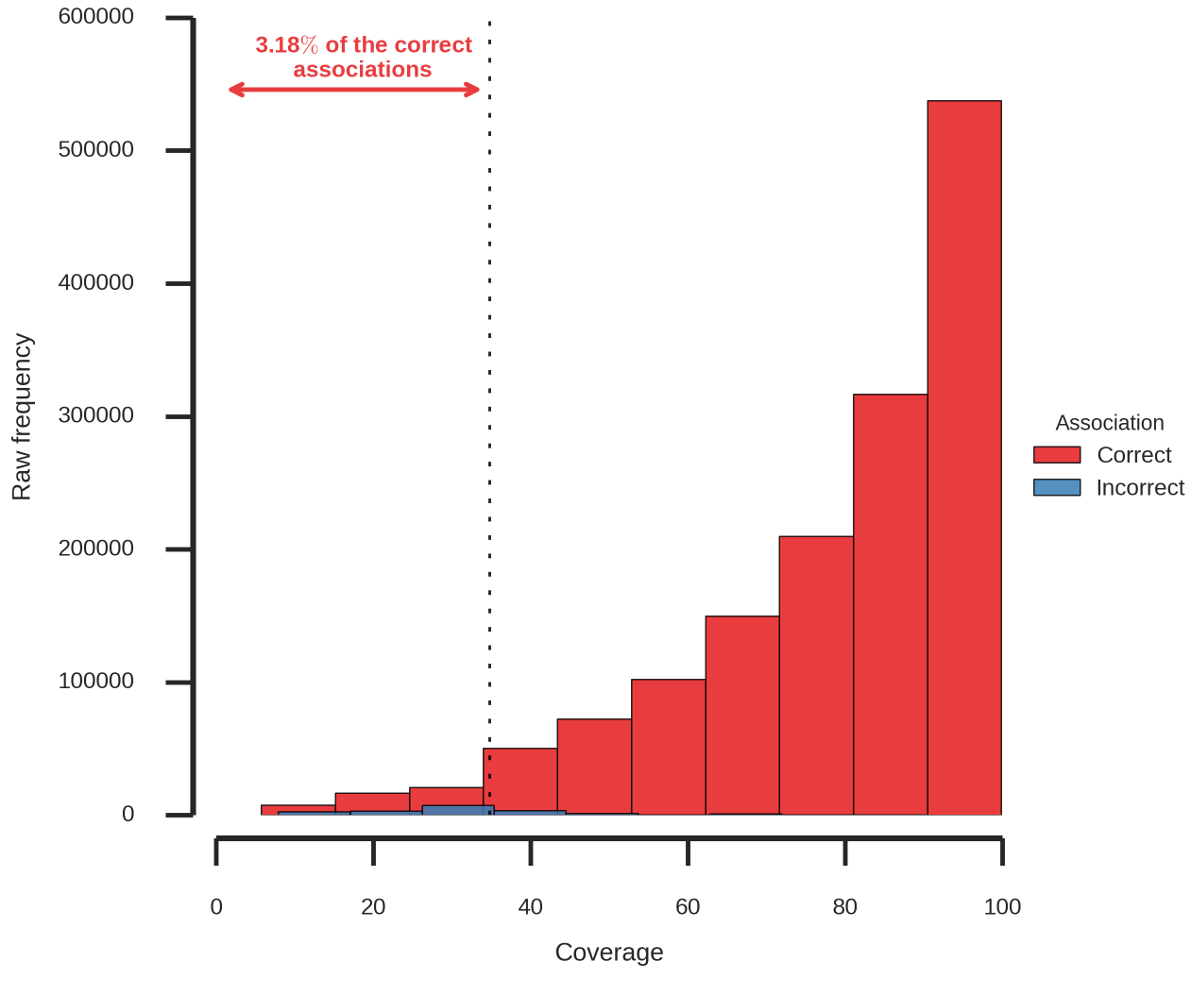

Supplement: Supplementary file 2 — Figure S1. The frequency distribution of sequence query coverage for correct and incorrect fold associations: “Blue” represents the incorrect and “red” the correct associations respectively. The median for the distribution of “incorrect” associations corresponds to 30.13% query coverage, represented by the dotted line. 3.18% of the correct fold associations are to the left of this median value. (PNG 72 kb) [file 13062_2018_209_MOESM2_ESM.png]

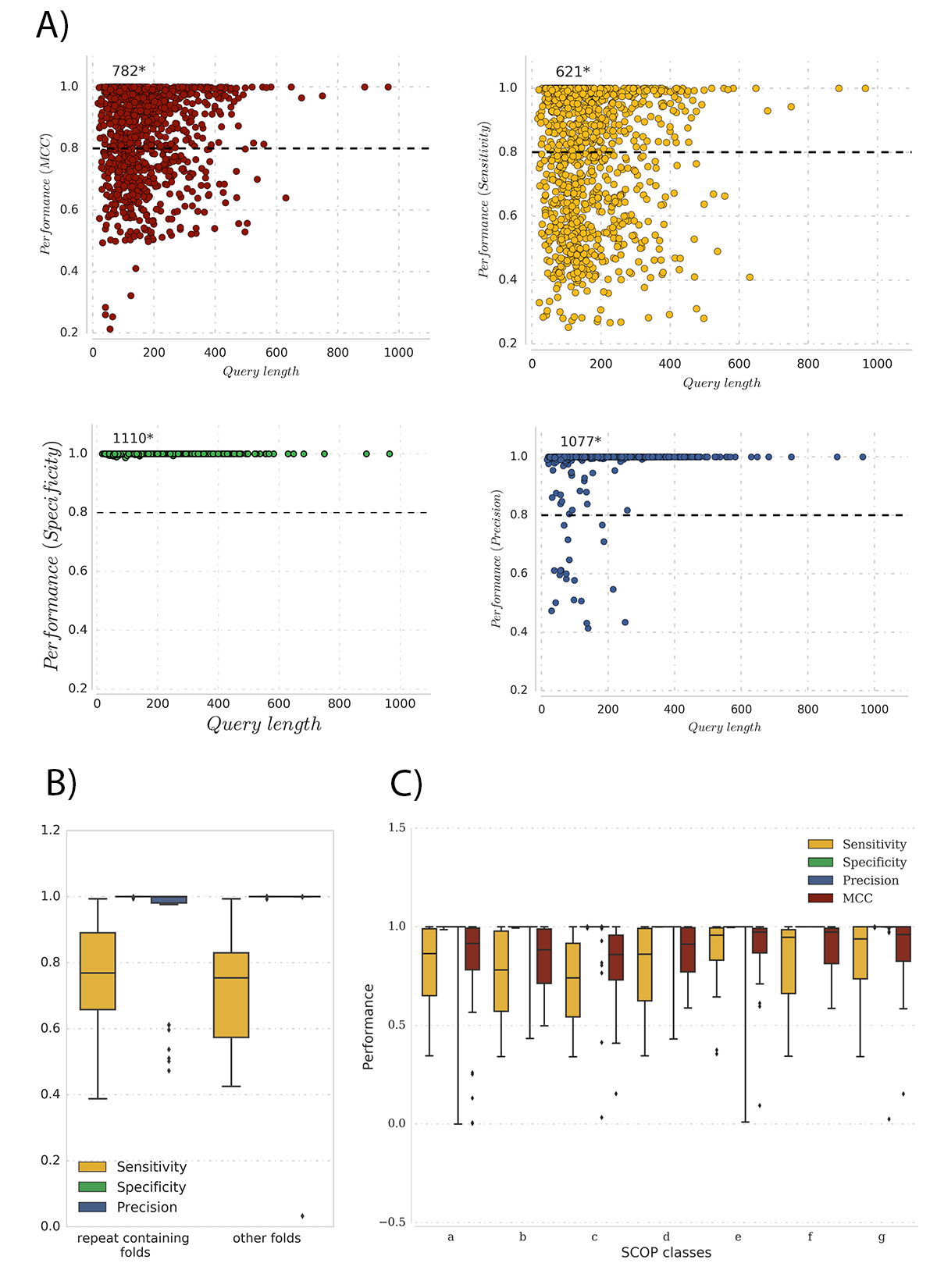

Supplement: Supplementary file 3 — Figure S2. Performance of our approach as a function of different parameters: a) Query length – Performance as a function of the number of amino acids, annotated are the points above 0.8 for: Sensitivity (621*), Specificity (1110*), Precision (1077*) and MCC (782*). b) Repeat containing folds – Comparative performance of folds in our assessment dataset containing structural repeats with other folds. c) Secondary structure based SCOP classes – Performance metrics evaluated across different secondary structure based SCOP classes “a” through “g”, which are as follows: a (All-α), b (All-β), c (α/β), d (α+β), e (Multi-domain proteins), f (Membrane and cell surface proteins and peptides) and g (Small proteins). (PNG 395 kb) [file 13062_2018_209_MOESM3_ESM.png]

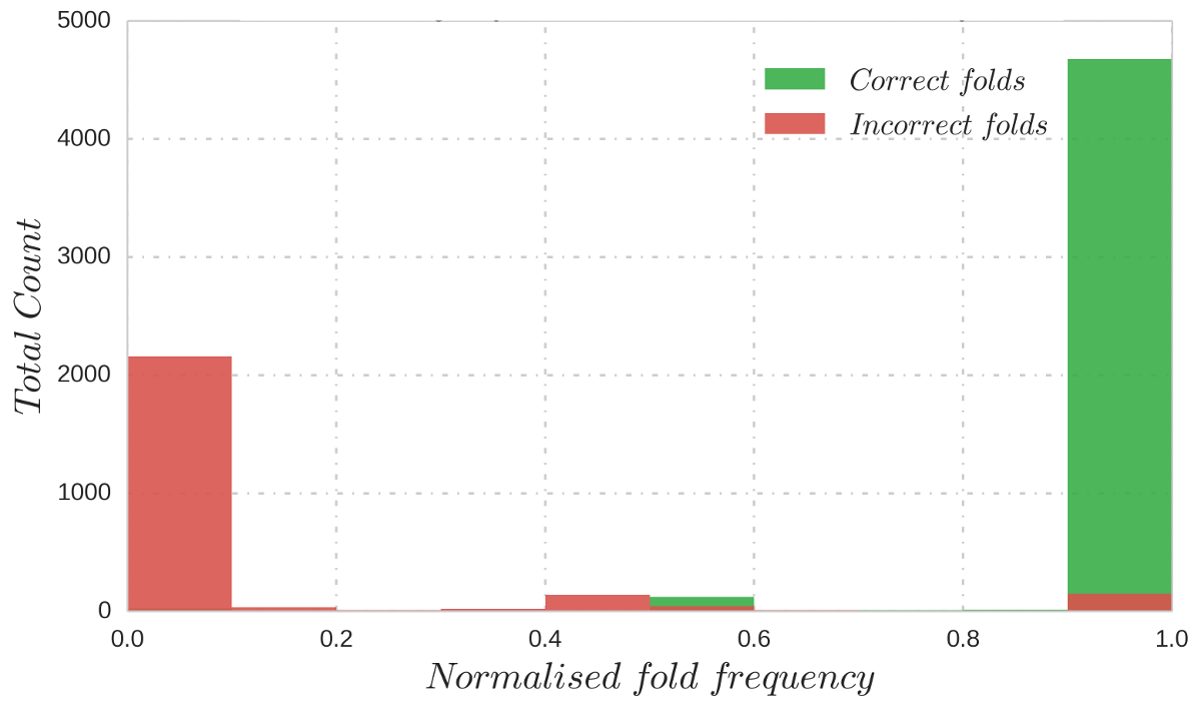

Supplement: Supplementary file 5 — Figure S3. The normalized fold frequency of correct vs. incorrect associations for the assessment dataset: The preponderance of ‘correct’ associated folds (in green) is observed at a higher normalized fold frequency than other ‘incorrect’ fold associations (in red). (PNG 92 kb) [file 13062_2018_209_MOESM5_ESM.png]

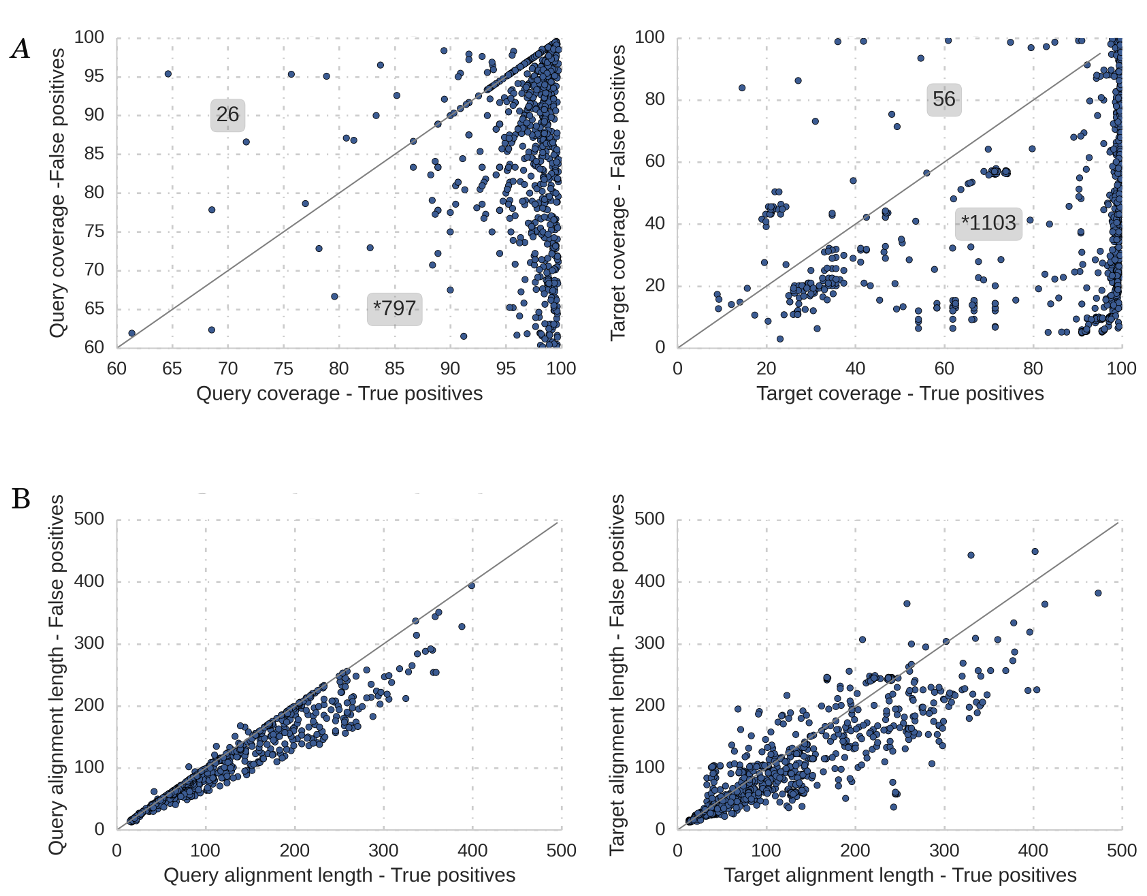

Supplement: Supplementary file 6 — Figure S4. False vs. True positives for queries in the assessment dataset: The distribution of true positives vs. false positives as a function of a) Query and target coverage. b) Query and target alignment length (number of residues in the alignment). (PNG 363 kb) [file 13062_2018_209_MOESM6_ESM.png]
